# Depression as a risk factor for adverse outcomes in coronary heart disease

**DOI:** 10.1186/1741-7015-11-131

**Published:** 2013-05-15

**Authors:** Kenneth E Freedland, Robert M Carney

**Affiliations:** 1 Department of Psychiatry, Washington University School of Medicine, 4320 Forest Park Avenue, Suite 301, St Louis, Missouri 63108, USA

**Keywords:** Acute coronary syndrome, Antidepressive agents, Coronary disease, Depression, Depressive disorder, Mortality, Myocardial infarction, Psychotherapy

## Abstract

**Background:**

Depression is firmly established as an independent predictor of mortality and cardiac morbidity in patients with coronary heart disease (CHD). However, it has been difficult to determine whether it is a causal risk factor, and whether treatment of depression can improve cardiac outcomes. In addition, research on biobehavioral mechanisms has not yet produced a definitive causal model of the relationship between depression and cardiac outcomes.

**Discussion:**

Key challenges in this line of research concern the measurement of depression, the definition and relevance of certain subtypes of depression, the temporal relationship between depression and CHD, underlying biobehavioral mechanisms, and depression treatment efficacy.

**Summary:**

This article examines some of the methodological challenges that will have to be overcome in order to determine whether depression should be regarded as a key target of secondary prevention in CHD.

## Background

The past three decades of research have produced compelling evidence that depression is a risk factor for mortality and cardiac morbidity in patients with coronary heart disease (CHD) [1-5]. Nevertheless, many questions about this phenomenon have not yet been answered unequivocally, and skeptics still have some legitimate grounds for their skepticism.

The most important scientific questions about depression in patients with CHD are whether it is not just a risk marker but a causal risk factor for adverse CHD outcomes [6], and which biobehavioral mechanisms, among the many that have been proposed, link depression to these outcomes [7,8]. The most important clinical questions are whether depression is modifiable (that is, treatable) in patients with CHD, and whether its treatment improves cardiac event-free survival [9].

This article explains some of the principal reasons why it has been difficult to answer these questions to a high degree of certainty. It discusses methodological challenges, observational research, randomized controlled trials (RCTs), and other treatment-related research. It also highlights an interesting paradox that has recently emerged in the treatment literature, and discusses its implication for future research.

## Discussion

### Defining and measuring depression

Several long-standing controversies have surrounded the definition and measurement of depression in patients with CHD. The central question that drives these controversies is whether features that resemble depression represent ‘real’ depression in these patients. This question is not exclusive to CHD; it also casts doubt on the diagnostic validity of depression in the presence of other chronic medical illnesses [10]. One of the reasons is that some of the symptoms of depression are non-specific; that is, they can also be symptoms of other disorders [11]. Fatigue is a good example. In a patient with CHD, it might be due to depression, but it could also be due to CHD, medical comorbidities, side effects of medications, or deconditioning. One way to handle non-specific symptoms is to assume that they are not due to depression and to omit them from screening instruments and diagnostic interviews [12]. This approach is problematic, for several reasons. First, in many cases, these symptoms may be due partially or entirely to depression, and it is rarely possible to pinpoint their etiology. Second, there are fairly strong correlations, even in medically ill patient populations, between non-specific symptoms such as fatigue and less ambiguous cognitive and emotional symptoms of depression such as dysphoric mood, feelings of worthlessness, and excessive or inappropriate guilt [13,14]. Finally, the American Psychiatric Association’s *Diagnostic and Statistical Manual of Mental Disorders*, fourth edition (DSM-IV) criteria for depressive disorders [15] indicate that features such as fatigue should be counted as symptoms of depression unless they are ‘…due to the direct physiological effects of a substance (for example, a drug of abuse, a medication) or a general medical condition (for example, hypothyroidism)’. In other words, unless there is clear evidence that a symptom is entirely due to the direct, physiological effects of a medical condition or medication, it should be counted towards the diagnosis of depression.

Giving the benefit of the doubt to non-specific symptoms may increase the risk of false positive diagnoses of depression. However, the DSM-IV criteria for major depression reduce this risk by requiring (1) the presence of at least one cardinal symptom (dysphoric mood and/or pervasive loss of interest or pleasure in usual activities) plus four or five additional symptoms from a list of nine distinguishing features of depression, (2) the symptoms must be present most of the day nearly every day, (3) the symptoms must have been present for at least 2 weeks, and (4) there should be evidence that the symptoms are causing distress and/or impairment in one or more domains of daily functioning.

The cognitive and emotional symptoms of depression in cardiac patients are sometimes disregarded as well, but for a very different reason: The importance of these symptoms is minimized because they are ‘understandable’, as in, ‘Of course he’s feeling down, he just had a heart attack’ [16]. This view is unjustified. If a depressive episode is precipitated by a stressful event, regardless of whether it is a myocardial infarction (MI) or any other kind of adversity, it is still a depressive episode. Relatively severe and/or persistent cases of depression are clinically significant regardless of whether their etiology is ‘understandable’ or obscure. In addition, it is a mistake to assume that if a patient is found to be depressed after an adverse event such as an MI, the depression is necessarily due to that event. Many patients have a myocardial infarction during a depressive episode that may have started weeks or months before the cardiac event [17]. Furthermore, many patients start having depressive episodes years or even decades before there are any clinical manifestations of CHD [18].

These controversies would have succumbed long ago if there were a definitive diagnostic test for depression or if it were a monogenic disorder, but depression is a complex, multifactorial condition, and there is no definitive laboratory test for it. The research in this area is further complicated by a multiplicity of measures of depression. Some of the most frequently used instruments are listed in Table 1. The list is limited to measures that were recommended for use in research on depression and CHD in a National Heart, Lung, and Blood working group report, and that have been used in multiple studies of cardiac patients [19].

**Table 1:** Some widely used measures of depression in research on coronary heart disease (CHD)

**Format**	**Instruments and reference**
Self-report questionnaire	Patient Health Questionnaire (PHQ) [20-22]
	Beck Depression Inventory, first edition (BDI) [23]
	Beck Depression Inventory, second edition (BDI-II) [24]
Structured interview	Composite International Diagnostic Interview (CIDI) [25]
	Depression Interview and Structured Hamilton (DISH) [26]

In retrospective studies of large healthcare databases, depression is typically defined by clinical diagnosis and/or antidepressant prescriptions. Despite the fact that these sorts of studies are plagued by classification errors, some of them have found significant associations between depression and adverse outcomes in cardiac patients, for example [27,28].

In many prospective studies, depression has been measured by self-report questionnaires [17,29,30]. The total score can be used to characterize the overall severity of depressive symptoms. Cutoff scores can be used to differentiate between non-depressed and depressed patients, and scores in the depressed range may be subdivided into mild, moderate, and severe levels of depression. Several different questionnaires have been used to study depression in cardiac patients, despite the fact that there are differences in their predictive values [31-34]. In other prospective studies, depression is defined according to the DSM-IV or *International Classification of Diseases*, ninth revision (ICD-9) criteria for depressive disorders [35-37]. The symptoms of depressive disorders are usually evaluated in these studies by a structured interview rather than by a self-report questionnaire [35,36,38]. Some studies suggest that interview-based depression diagnoses have greater predictive value than questionnaires vis-à-vis cardiac outcomes; other studies suggest the opposite. The jury is still out on this question, but it is clear that differences among measurement methods help to explain differences among studies as to whether and how strongly depression predicts cardiac outcomes [39,40].

### Subtypes of depression

Depression is a polythetic syndrome in which different patients present with different combinations of symptoms [41]. For example, sleep disturbance is a prominent feature of depression in some cases but not in others [42]. The phenotypic complexity of depression has created interest in the question of whether particular symptoms of depression, or clusters of symptoms, are associated with a higher risk of adverse cardiac outcomes than are other symptoms or symptom clusters. Although there are a number of different lines along which the syndrome of depression has been carved, the cognitive/somatic distinction is the one that has gained the most attention in the behavioral cardiology literature [43-48].

Research on the cognitive and somatic features of depression in cardiac patients can be difficult to interpret, for several reasons. First, there several different ways to group or cluster these symptoms, including various forms of statistical factor analysis and ‘face validity’ approaches. Different methods can and do result in different groupings [43-47,49-53]. Second, many studies have shown that there is a moderately strong correlation between the cognitive and the somatic symptoms of depression, and that both sets of symptoms tend to rise and fall along with the overall severity of depression. The cognitive and somatic symptoms of depression are probably manifestations of a single polythetic disorder, not indicators of two distinctly different disorders [13,14]. Finally, studies that have compared the predictive values of cognitive and somatic symptoms have produced conflicting results. Some studies of patients with coronary disease have shown that somatic symptoms are better predictors of cardiac outcomes than are cognitive symptoms, but other studies have not found this difference [44,49,54]. Studies of other cardiac patient populations have increased the heterogeneity of the findings in this area. For example, studies of depression after coronary artery bypass graft (CABG) surgery shown that cognitive symptoms are better predictors than are somatic symptoms, the very opposite of the pattern found in some studies of post-acute coronary syndrome (ACS) patients [55,56].

### Time factors

Coronary atherosclerosis usually starts to develop long before it produces any clinical manifestations or requires intervention. Its initial clinical presentation is often lethal, and relatively little is known about the role of depression in these cases. Thus, most of what we know about the effect of depression on cardiac outcomes is based on studies of patients who have survived the clinical onset of CHD. Whether the strength of the effect of depression differs between patients who survive the onset of CHD and those who do not is very difficult to study.

Initial, non-lethal presentations of coronary disease come in a variety of forms, including discovery via routine testing, exertional angina, unstable angina, acute MI, and others, and they may or may not require coronary revascularization via percutaneous intervention (PCI) or CABG surgery. Some depression studies enroll patients after a particular kind of initial presentation, such as after an acute MI. Other studies are more inclusive and enroll patients who began their cardiac careers in a variety of different ways. In some studies, depression is assessed shortly (for example, within 1 month) after one of these initial cardiac events. In other studies, the first assessment of depression occurs long after (for example, 3 to 6 months) the initial cardiac event. In still other studies, the first assessment of depression may not follow the initial cardiac event, at least not in every case. For example, in a study of depression in patients with ‘stable CHD’, some of the patients may have no history of acute MI, others may have had only one MI, and still others may have had multiple MIs.

Depression is a complex condition in terms of its timing. General population studies (for example, [50]) have found that typically, the first episode of major depression occurs in childhood, adolescence, or young adulthood, but that in some cases, it occurs in middle or old age. The chronicity of major depressive episodes is also highly variable; they typically last a few weeks or months, but some last for years. In addition, there are substantial differences in the number of depressive episodes that individuals experience over their lifetime. Whereas some people have an initial episode and never have another, others go on to have multiple episodes [57,58]. The number of episodes an individual has had depends, in part, on the age at which he or she is assessed. Since the initial clinical presentation of CHD usually occurs after age 50, many patients have had one or more prior episodes of depression by the time they are enrolled in a study, whether or not they happen to be depressed at the time of enrollment.

It is often difficult for patients to give a reliable, accurate description of their current or recent depression symptoms. A patient might remember having felt fatigued recently, but be unable to accurately recall when it started or how frequently it has been present over the past 2 weeks. It can be even more difficult to remember depressive episodes that occurred years or even decades ago, and patient’s recall of such remote events can be biased by many different factors, including their current mood state and medical condition. This can be especially problematic when patients are assessed shortly after a stressful cardiac event, for example, while hospitalized for an acute MI [59,60]. Inaccurate recall and reporting biases may contribute to the broader problem of underdiagnosis of depression in patients with heart disease [11,61,62].

All of these factors make it difficult to pinpoint the temporal relationship between depression and CHD. Both conditions are ‘moving targets’, and it can be difficult for patients to remember and report relevant details. This is unfortunate because the temporal relationship between these two conditions is important, for a variety of reasons. One is that in some prospective studies, depression is assessed only once (for example, after an acute MI), but recurrent cardiac events and deaths are ascertained over a follow-up period of up to several years. If depression predicts cardiac outcomes in such a study, it is not clear whether lasting harm was caused by the depressive episode that occurred around the index event. An alternative possibility is that the patients who were depressed at that time also tend to be depressed at other times during the follow-up. It may be that depression only poses a risk if it is present around the time of a recurrent cardiac event, and not only around the time of the index event. There is no way to differentiate between these possibilities in studies that only assess depressed after the index event.

In other prospective studies, depression is assessed periodically during the follow-up period. This is an improvement over single assessments. However, if depression is measured at very wide intervals (for example, once a year), most of the cardiac events will occur between measurements. Consequently, there will still be uncertainty as to whether patients are at risk for recurrent cardiac events only during depressive episodes, or if they remain at increased risk after or between depressive episodes.

The temporal relationship between depression and CHD may be important for other reasons as well. For example, some studies suggest that patients who have their first ever depressive episode around the time of an acute MI are at higher risk for adverse outcomes than are patients who are equally depressed but who have also had prior episodes of depression [18,63]. It could be, however, that among currently depressed MI patients, the ones who are at the highest risk are also happen to be the most likely to forget that they have had previous episodes of depression. Another possibility is that vascular disease may contribute both to cardiovascular events and to late-life depression [64,65]. As another example, some studies suggest that the prognostic importance of a depressive episode depends on whether its onset precedes or follows an acute coronary event [66]. Biased recall is a significant challenge in these studies, since the assessment of depression invariably occurs only after the index cardiac event.

### Causal models and mechanisms

If depression predicts worse outcomes in patients with CHD (and the evidence strongly suggests that it does), then it is important to find out why. What connects these two very different conditions with one another?

Different causal models of the relationship between depression and CHD give rise to interest in different mechanisms. The model that has captivated most of the researchers in this field posits that depression plays a causal role in adverse cardiac outcomes. In this model, the adverse effects of depression on cardiac outcomes may be mediated by behavioral factors, biological factors, or both. The leading candidates on the behavioral side are factors such as physical inactivity [67], smoking [68], and non-compliance with cardiac medications [69-72]. On the biological side, the candidates that have received the most attention are cardiovascular autonomic dysregulation [73-75] and inflammation [76-83]. Depression is associated with all of these phenomena. On average, for example, depressed patients tend to have higher resting heart rates and lower heart rate variability than otherwise similar non-depressed patients. They are also more likely to smoke and less likely to engage in regular exercise and take their cardiac medications as prescribed. The addition of these factors to survival analysis models often attenuates the effect of depression on cardiac event-free survival. Thus, they might turn out to be the mechanistic linkages that explain why depressed cardiac patients are at higher risk of adverse outcomes. The expense and practical difficulty of measuring some of these biobehavioral factors, especially in large studies with lengthy follow-up periods, has impeded progress toward a definitive mechanistic model [8].

An alternative causal model posits that comorbid depression in CHD is caused by the heart disease [84]. This model assumes that the apparent effect of depression on cardiac outcomes is an epiphenomenon, that is, the patients with the most severe cases of CHD tend to have the worst outcomes and tend to be the ones who get depressed. The discussion of time factors (above) provides some reasons to question the premise that depression in patients with CHD is necessarily due to their CHD. Nevertheless, it remains possible that the patients who are the most severely depressed also tend to be the ones with the most severe CHD [4].

There are a number of ways to characterize the anatomical or pathophysiological severity of various aspects of CHD. For example, some studies have used Gensini scores to characterize the number of stenotic coronary artery segments, or cardiac enzyme levels to measure the severity of acute MI. Few studies have found that these sorts of measures correlate very strongly (if at all) with the presence or severity of depression in patients with CHD. In addition, quite a few prognostic studies have controlled for these indicators and have nevertheless found significant associations between depression and cardiac outcomes [2,39,85].

However, measures of the symptomatic or functional severity of heart disease do correlate with depression [86-89], and there is substantial evidence that depression contributes to functional impairment and lowers symptom-reporting thresholds, both in CHD and in other patient populations as well [90,91]. Thus, controlling for indicators of the symptomatic or functional severity of heart disease creates a ‘chicken and egg’ problem, and thereby contributes little to our understanding of the causal relationship between depression and adverse cardiac outcomes.

A third causal model posits that the association between depression and CHD is due to shared heritability. Studies that have tested this model have found evidence of shared heritability [84,92,93]. However, there is also evidence that among twins at high genetic risk for both disorders, the risk of developing ischemic heart disease is significantly higher in those with than without phenotypic expression of depression [94]. This suggests that both shared genetic liability and exposure to depression play a role, and that the relationship between depression and CHD is not an epiphenomenon.

These models are not mutually exclusive. The burden of CHD (along with other chronic illnesses that are common in patients with CHD) may promote depression, exposure to depression may promote adverse cardiac outcomes, and shared genetic factors may predispose some individuals to both conditions.

### Treatment research

Two complementary goals have motivated research on the treatment of depression in patients with CHD. One is to identify treatments that are both safe and effective for depression in this patient population, and the other is to determine whether effective treatment of depression improves cardiac outcomes. The latter goal has pragmatic implications for clinical care, but it also important with respect to the question of whether depression plays a causal role in adverse cardiac outcomes.

Cardiac patients were seldom treated for depression prior to the development of selective serotonin reuptake inhibitors (SSRIs), because (among other reasons) the only antidepressants that were available at the time were cardiotoxic. Most of the recent studies of antidepressant medications for depression in cardiac patients have evaluated SSRIs such as sertraline or citalopram. The weight of the available evidence indicates that these agents are relatively safe for patients with CHD but that their efficacy is less than impressive [95-99].

The Sertraline Antidepressant Heart Attack Randomized Trial (SADHART) was one of the largest and most rigorous studies in this area. A total of 369 patients with major depression were enrolled within 1 month of an acute coronary event and randomly assigned to receive sertraline or placebo for 24 weeks. The safety results were favorable, but post-treatment scores on the Hamilton Rating Scale for Depression (HRSD) did not differ between the groups. There were significant differences on the HRSD within the subgroup of patients with severe depression, as defined by a HRSD score of 18 or higher at baseline, but not in the patients with less severe major depression. Even in the severe subgroup, the post-treatment HRSD scores of the sertraline and placebo groups differed by less than 3 points [95].

Trials that have tested psychotherapeutic interventions, or combinations of psychotherapy and medication, have also produced mixed results. The Enhancing Recovery in Coronary Heart Disease (ENRICHD) study is the largest trial to date in this area. A total of 2,481 patients were recruited within 1 month of an acute MI and met the study’s criteria for depression (n = 978), low perceived social support (n = 647), or both (n = 856). (Low perceived social support was an eligibility criterion for ENRICHD because, like depression, it increases the risk of adverse cardiac outcomes). The participants were randomly assigned to cognitive behavior therapy (CBT) or to usual care (UC). Some patients in the intervention arm received sertraline in addition to CBT. The intervention was superior to usual care for depression among the depressed participants, but the effect was modest; on average, the groups differed by less than 2 points on the HRSD and less than 3 points on the Beck Depression Inventory (BDI). The intervention also had statistically significant but small effects on social support among the participants who had low perceived social support at enrollment [100].

There was no difference between the treatment and control groups in cardiac event-free survival. However, ENRICHD provided a weak test of the causal risk factor hypothesis because the ENRICHD intervention had weak effects on depression and social support. It will not be possible to conduct a much stronger test of this hypothesis until more effective interventions for depression are developed. There has been some progress toward this objective [101], but we may have to wait a few more years for a rigorous, randomized, controlled test of the hypothesis that depression is a modifiable, causal risk factor for adverse cardiac outcomes in patients with CHD.

Several trials that have produced disappointing primary results have turned out to be very informative in an unexpected way. Secondary analyses of these trials have revealed that patients who remain depressed despite aggressive treatment are at significantly higher risk for adverse cardiac outcomes than are patients who do respond to treatment. Treatment-resistant depression appears to be a particularly high-risk form of depression in patients with CHD, and a more robust predictor of adverse cardiac outcomes than other subtypes, such as depression with salient somatic symptoms [9].

This line of research has been productive, but its implications are disconcerting. As discussed above, the overarching goal of this entire area of research has been to determine whether depression is both modifiable and causally related to adverse cardiac outcomes. Treatment-resistant depression may be a causal risk factor for cardiac morbidity and mortality, but until we find more treatments that are efficacious this form of depression is clearly not modifiable. Treatment-resistant depression is a common problem both in otherwise medically well psychiatric patients and in patients with chronic medical comorbidities. However, coronary disease and other medical comorbidities may contribute to depression treatment resistance [102,103]. The Sequenced Treatment Alternatives to Relieve Depression (STAR*D) trial and other recent studies have shown that stepped care algorithms and non-pharmacological interventions can improve depression in many cases that do not respond to first-line treatments, but that complete remission of depression is an elusive goal in many of these cases [104-107]. Identification of more efficacious interventions for treatment-resistant depression is a high priority for research on depression in general, and in patients with coronary disease in particular.

## Summary

Numerous studies and meta-analyses have shown that depression is a robust predictor of adverse outcomes in CHD. However, it has been difficult to determine whether depression plays a causal role in these outcomes, as well as the biobehavioral pathways that link depression to cardiac morbidity and mortality. It has also been difficult to determine whether effective treatment of depression can improve cardiac outcomes, primarily because highly effective treatments are not yet available. Depression is a common comorbid condition in CHD, and there is growing recognition of the harm it can cause. It is difficult to answer some of the key questions about depression in patients with CHD, but it is worth the effort to try. Further research is needed to identify significant moderators of depression treatment outcomes, and to develop efficacious interventions for treatment-resistant depression. Such studies could improve the clinical care of patients with CHD, while helping to resolve the question of whether depression is a causal risk factor for cardiovascular morbidity and mortality in these patients.

## Abbreviations

ACS: Acute coronary syndrome; BDI: Beck Depression Inventory; CABG: Coronary artery bypass graft; CHD: Coronary heart disease; CIDI: Composite International Diagnostic Interview; DISH: Depression Interview and Structured Hamilton; DSM-IV: Diagnostic and Statistical Manual, 4^th^ edition; ENRICHD: Enhancing Recovery in Coronary Heart Disease; HRSD: Hamilton Rating Scale for Depression; ICD-9: International Classification of Diseases, 9^th^ edition; MI: Myocardial infarction; PCI: Percutaneous coronary intervention; PHQ: Patient Health Questionnaire; RCT: Randomized controlled trial; SADHART: Sertraline Antidepressant Heart Attack Randomized Trial; SSRI: Selective serotonin reuptake inhibitor; STAR*D: Sequenced Treatment Alternatives to Relieve Depression; UC: Usual care.

## Competing interests

The authors declare that they have no competing interests.

## Authors’ contributions

Both KEF and RMC contributed equally to the conception, writing, and revision of this manuscript. All authors read and approved the final manuscript.

## Authors’ information

Kenneth E. Freedland, Ph.D. is a Professor of Psychiatry and Psychology and the Associate Director of the Behavioral Medicine Center at Washington University School of Medicine in St. Louis, Missouri, USA. Robert M. Carney, Ph.D. is a Professor of Psychiatry and Psychology and the Director of the Behavioral Medicine Center at Washington University School of Medicine in St. Louis, Missouri, USA. Drs. Freedland and Carney have been collaborating since 1986 on research on the role and treatment of depression in patients with coronary heart disease or congestive heart failure.

## Pre-publication history

The pre-publication history for this paper can be accessed here:

http://www.biomedcentral.com/1741-7015/11/131/prepub
